# Feasibility of strain-encoded magnetic resonance at 0.55T

**DOI:** 10.1016/j.jocmr.2025.101870

**Published:** 2025-02-25

**Authors:** John L. Heyniger, Yingmin Liu, Nikita Nair, Preethi Chandrasekaran, Katherine Binzel, Vinay Kumar, Shyam S. Bansal, Donel Tani, Farouk Osman, Vedat O. Yildiz, Juliet Varghese, Yuchi Han, Orlando P. Simonetti

**Affiliations:** a The Ohio State University College of Medicine, Columbus, Ohio, USA; b The Ohio State University, Davis Heart and Lung Research Institute, Columbus, Ohio, USA; c Pennsylvania State University Heart and Vascular Institute, Hershey Medical Center, Hershey, Pennsylvania, USA; d Myocardial Solutions, Morrisville, North Carolina, USA; e Center for Biostatistics, The Ohio State University College of Medicine, Columbus, Ohio, USA; f The Ohio State University, Department of Biomedical Engineering, Columbus, Ohio, USA; g The Ohio State University, Department of Internal Medicine, Division of Cardiovascular Medicine, Columbus, Ohio, USA; h The Ohio State University, Department of Radiology, Columbus, Ohio, USA

**Keywords:** Cardiovascular magnetic resonance (CMR), Strain-encoded magnetic resonance (SENC), Low-field MRI

## Abstract

**Background:**

Low-field (<1.0T) wide-bore cardiovascular magnetic resonance (CMR) has the potential to improve patient accessibility; however intrinsically reduced signal-to-noise ratio may affect techniques such as strain-encoded magnetic resonance (SENC), a method to quantify regional strain. We sought to characterize the performance of SENC on a low-field system in a phantom, healthy subjects, and a porcine model of myocardial infarction (MI).

**Methods:**

A prototype SENC sequence was implemented on 0.55T and 1.5T systems and used to scan a phantom and 16 healthy volunteers. 10 subjects underwent repeat scans at each field strength for scan-rescan repeatability testing. T-tests were used to compare global strain values; *reproducibility* between field strengths and scan-rescan *repeatability* were assessed via Bland-Altman and intra-class correlation (ICC). Adjunctive SENC followed by late gadolinium enhancement (LGE) was acquired at 0.55T in a porcine MI model (n = 6). Left ventricular (LV) segments were categorized by LGE, and segmental strain was compared via one-way analysis of variance.

**Results:**

Phantom strain showed no significant differences between field strengths (p > 0.10). In volunteers, mean LV global longitudinal (GLS) and circumferential strain (GCS) were -19.4% ±1.1 and -20.4% ± 0.9 at 0.55T compared to -18.7 ±1.4% and -19.2% ±1.6 at 1.5T (p > 0.10). LS proved to have better agreement than CS, and mean biases were low for both global and segmental comparisons throughout. Limits of agreement were good for global strain but wider for segmental measurements. Pooled LV segmental strain ICC showed good reproducibility for LS between field strengths (0.78) and good repeatability at 0.55T (0.89); however, reproducibility for CS was fair (0.60), as was repeatability at 0.55T (0.64). In the porcine infarct model, segmental LS in LGE+ segments (-10.8% ±4.0) was less negative than remote segments (-16.8% ± 5.1), p < 0.001. Similarly, segmental CS in LGE+ vs remote segments was -11.9% ± 2.7 vs -14.6% ± 2.7; p = 0.0011.

**Conclusion:**

Our results support the feasibility of SENC at 0.55T, with accurate phantom measurements, good agreement of global values with 1.5T in human volunteers, and correlates of functional impairment with known MI. Reproducibility showed minimal systemic bias but at times substantial limits of agreement. Repeatability of global and segmental LS at 0.55T was similar to established 1.5T performance, although CS was notably worse than LS. LV CS may lack sufficient reliability in its current implementation for use at 0.55T.

## Graphical abstract

**Figure:**
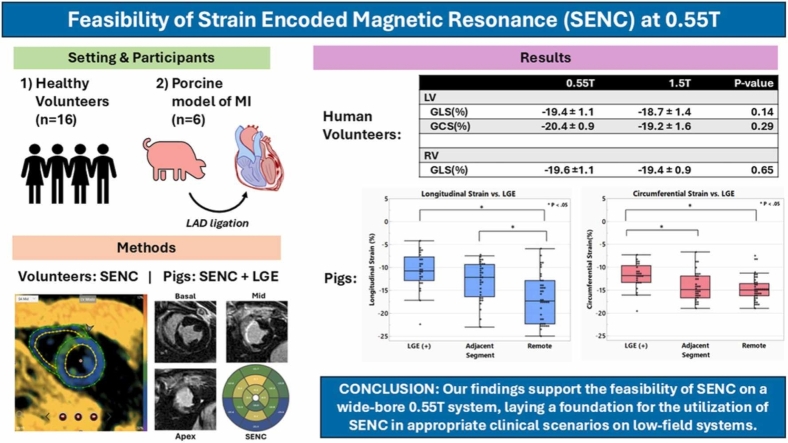

## 1 Introduction

Cardiovascular magnetic resonance (CMR) is a powerful tool for assessing cardiovascular structure, function, myocardial tissue characterization, and blood flow. However, its high costs limit its use, particularly in lower-resource areas. Recently, low-field (<1.0T) CMR has gained attention for its potential to deliver diagnostic-quality images at reduced costs, with the potential to improve accessibility in underserved regions [1], [2]. Furthermore, low-field systems with larger bore diameters can accommodate patients with claustrophobia and severe obesity, the latter expected to comprise nearly 25% of the United States adult population by 2030 [3]. The reduced signal-to-noise ratio (SNR) at low field requires that cardiac imaging sequences be optimized and validated before clinical use [4].

Strain-encoded magnetic resonance (SENC) imaging is a reproducible CMR technique [5] for measuring left ventricular (LV) and right ventricular (RV) strain [6], sensitive to subtle changes in cardiac function. SENC encodes strain perpendicular to the imaging plane, measuring longitudinal strain (LS) and circumferential strain (CS) both globally (GLS/GCS) and segmentally throughout the cardiac cycle. When compared to feature tracking (FT), which estimates strain using conventional cine images, SENC has shown greater segmental strain reproducibility [7] and higher sensitivity and specificity for detecting segmental wall motion abnormalities associated with myocardial infarction (MI) [8].

While the feasibility of standard CMR sequences on low-field systems has been demonstrated [4], [9], [10], SENC performance at lower fields has not been explored. We sought to investigate SENC performance at 0.55T by first confirming accuracy in a dynamic compression phantom and then evaluating scan-rescan repeatability and head-to-head agreement with SENC data acquired at 1.5T in healthy human subjects. In addition, we performed SENC imaging at 0.55T in an ongoing porcine study of MI to evaluate the capability to detect impaired strain in known infarcted myocardial segments.

## 2 Methods

### 2.1 SENC pulse sequence

All experiments were performed on an 80-cm bore commercial 0.55T system and a 70-cm bore 1.5T system (MAGNETOM Free.Max and Sola, Siemens Healthcare, Forchheim, Germany). Maximum gradient amplitude and slew rates were 26 mT/m and 45 T/m/s for 0.55T compared to 45 mT/m and 200 T/m/s for 1.5T. Briefly, the SENC technique tags the myocardium across the slice thickness, and the signal reflects compression or expansion of the tag lines with myocardial deformation. Similar to phase contrast velocity mapping, SENC encodes two sets of images to quantify the relative deformation (strain) in the through-plane (i.e., slice) direction. The largest and smallest expected strain values are used to specify “high tuning” and “low tuning” gradient pulses. A maximum signal is generated if the tissue deformation matches the encoded strain value. SENC is run as a multi-frame cine acquisition, with high and low tune acquisitions interleaved such that time-varying strain can be measured throughout the cardiac cycle. Given that SENC measures strain in the through-plane direction only, LS is measured in short-axis views, and CS in long-axis views; however, SENC is incapable of measuring radial strain. A segmented k-space spoiled gradient echo acquisition was used at both field strengths with matching spatial resolution. The SENC method imparts signal dephasing in the slice direction reducing overall SNR; therefore, to bolster SNR, a relatively thick slice of 12 mm was used for volunteer scans at both 1.5T and 0.55T, and a thickness of 15 mm was used for the pig scans at 0.55T. Receiver bandwidth was reduced from 600 Hz/pixel at 1.5T to 200 Hz/pixel at 0.55T to partially offset the reduced SNR at lower field. Shorter native myocardial T1 may have warranted the use of higher flip angles at 0.55T than at 1.5T, but this was not explored. The longer readout combined with slower gradient performance resulted in marginally reduced temporal resolution and somewhat longer scan times at 0.55T compared to 1.5T. The sequence parameters are listed in Table 1.

**Table 1:** Strain-encoded magnetic resonance (SENC) scan parameters.

	1.5T healthy volunteers	0.55T healthy volunteers	0.55T porcine-infarct
TE/TR (ms)	1.9/4.2	2.8/6.9	2.8/6.9
Slice thickness (mm)	12	12	15
Flip angle (degrees)	12	12	10
Acquisition window (ms)	600	600	450
FOV (mm)	420 × 250	420 × 250	380 × 238
Acquisition matrix	96 × 60	96 × 60	96 × 60
Temporal resolution (ms)	16.8	20.8	27.5
Parallel imaging	GRAPPA rate 2	GRAPPA rate 2	GRAPPA rate 2
Scan time (# heartbeats)	8	10	8
Segment size	4	3	4
Bandwidth (Hz/pixel)	600	200	200

*TE echo* time, *TR* repeticion time, *FOV* field-of-view*, GRAPPA* generalized autocalibrating partially parallel acquisition

### 2.2 Phantom imaging and assessment

**Fig. 1:**
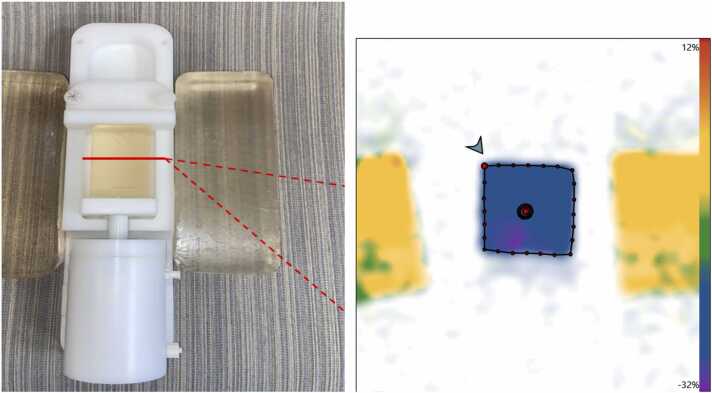
Left: A gel block (center) held in place by two flanking support blocks is deformed to a set strain by a pneumatic pump. One of two gel blocks can be used to provide either −20% or −30% known strain. Right: SENC strain is calculated in the through-plane direction using MyoStrain software. *SENC* strain-encoded magnetic resonance

A dynamic compression phantom was used to validate SENC accuracy at each field strength. Two silicone blocks were compressed using nominal strain values of −20% and −30%, respectively, at a cadence of 57 bpm. Generated strain was confirmed via manual measurement of the block dimensions at maximum extension and compression. SENC was acquired in the transverse plane to align the phantom deformation perpendicular to the slice plane (Fig. 1). SENC scans were run 10 times at each of the 2 deformation settings at each field strength. The phantom acquisition parameters were the same as used for human volunteers, shown in Table 1. All SENC images in this study were analyzed in MyoStrain (version 5.7, Myocardial Solutions Inc., Morrisville, North Carolina).

### 2.3 Human subjects acquisition and analysis

The investigation on healthy human subjects was approved by the local Institutional Review Board and all subjects provided informed consent. Sixteen healthy volunteers were scanned at both 0.55T and 1.5T to assess inter-scanner reproducibility; scan parameters are in Table 1. SENC images were acquired in short-axis base, middle, and apex views (for LS) as well as two-, three-, and four-chamber long-axis views (for CS). Ten of the 16 total volunteers underwent a scan-rescan protocol to assess repeatability at each field strength. Participants were scanned, slid out of the magnet bore, re-centered, and imaged again in the same session to assess repeatability. Strain was calculated from SENC images using user-defined endo- and epi-cardial contours and MyoStrain software. Global strains are automatically computed based on the peak of the average strain over the whole LV or RV myocardium.

### 2.4 Porcine model of myocardial infarction

SENC imaging was incorporated into an existing imaging protocol for an ongoing animal study of MI. The study was conducted with the approval of the local Institutional Animal Care and Use Committee. Briefly, the left anterior descending (LAD) artery was surgically ligated to induce non-reperfused MI in six Yorkshire pigs. At 8 weeks post-MI, the animals were scanned at 0.55T only. SENC images were acquired pre-contrast, and late gadolinium enhancement (LGE) images were acquired approximately 10 min after injection of 0.2 mmol/kg of gadobutrol (Gadavist, Bayer Healthcare, Whippany, New Jersey). The same sequence parameters (Table 1) and the same six imaging planes were used as described above. The extent of fibrosis was quantified from LGE in a 16-segment model using SuiteHeart (version 5.4, Neosoft, Pewaukee, Wisconsin). For each segment, the percentage area with LGE (0–100%) was measured and averaged over the six pigs to compare with strain findings. In a separate categorical analysis, each segment was classified as either presence of LGE (LGE+), adjacent to an LGE+ segment (adjacent), or neither (remote), and segmental LS and CS were compared between these categories.

### 2.5 Statistics

The central tendency is described as mean ± standard deviation. Phantom strain measurements as well as GLS and GCS findings in the volunteers were compared between field strengths using a paired *t*-test. Global and segmental strain values were assessed for both inter-scanner reproducibility (1.5T vs 0.55T) and scan-rescan repeatability within each field strength using Bland-Altman analysis. Longitudinal and circumferential segmental strains were additionally pooled together and assessed via Bland-Altman and intraclass correlation (ICC) analysis to evaluate the overall agreement in segmental measurements. The ICC analysis was a two-way model of absolute agreement. The variance of segmental strain repeatability at 1.5T vs 0.55T was compared with Levene’s test. To evaluate the relationship between strain measured at 0.55T and LGE in the pigs, segmental strain results were compared with LGE category using one-way analysis of variance with Tukey’s Honest Significant Difference post-hoc tests. Statistics were conducted in R Statistical Software (version v4.3.3; R Core Team 2023, R Foundation for Statistical Computing, Vienna, Austria) and JMP (version 16.0, SAS Institute Inc., Cary, North Carolina).

## 3 Results

Mean phantom strain at both preset deformations (−20% and −30%) showed no significant differences between field strengths (p > 0.10) (Table 2).

**Table 2:** Phantom imaging assessment at low field.

Phantom strain	Manually acquired strain	0.55T SENC	1.5T SENC	p value
−20%	−20%	−20.9% ± 1.8	−20.5% ± 0.5	0.47
−30%	−29%	−30.2% ± 0.4	−30.3% ± 0.2	0.56

Presented as mean (%) ± standard deviation

*SENC* strain-encoded magnetic resonance

The mean age of the 16 volunteers was 31 years (range 19–62 years); 10 were female. Average body mass index was 24.3 kg/m^2^. Mean LV and RV strain ranged from −18% to −21% on both systems (Table 3). GLS and GCS differences between field strengths were non-significant (p > 0.10). Reproducibility between field strengths and scan-rescan repeatability are presented on a global, pooled segmental, and segment-by-segment basis.

**Table 3:** Left ventricular (LV) and right ventricular (RV) global longitudinal strain (GLS) and global circumferential strain (GCS) results in 16 healthy volunteers.

	0.55T	1.5T	p value
LV			
GLS	−19.4±1.1	−18.7±1.4	0.14
GCS	−20.4±0.9	−19.2±1.6	0.29
RV			
GLS	−19.0±1.1	−19.2±1.2	0.71

Presented as mean (%) ± standard deviation

**Fig. 2:**
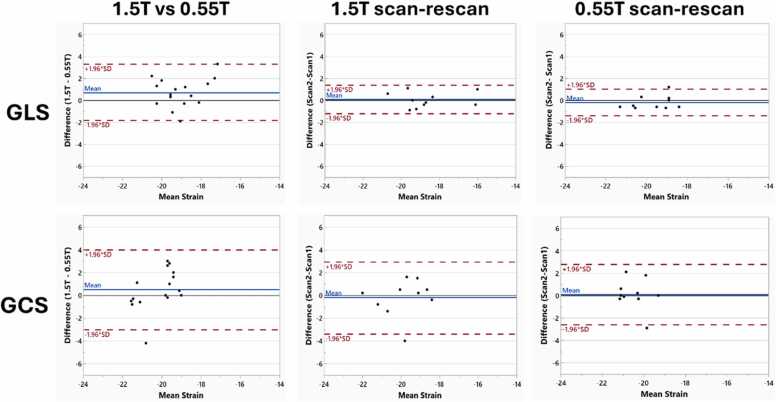
Left ventricular (LV) global longitudinal strain (GLS) and global circumferential strain (GCS) Bland-Altman analysis. Mean bias defined as 1.5T–0.55T for reproducibility between field strengths

Bland-Altman analysis of LV GLS and GCS showed small biases throughout, with wider limits of agreement (LoA) between field strengths compared to scan-rescan findings (Fig. 2). LS exhibited tighter LoA than CS throughout the study. Specifically, the LV GLS reproducibility between scanners showed a mean bias and LoA of +0.7 (−1.3, 3.3). Scan-rescan repeatability of LV GLS at 0.55T and 1.5T showed bias and LoA of +0.1 (−1.2, 1.3) and −0.2 (−1.4, 1.0), respectively. The reproducibility of LV GCS between scanners showed a mean bias and LoA of +0.5 (−3.0, 4.0), while scan-rescan repeatability at 1.5T and 0.55T showed bias and LoA of −0.2 (−3.4, 2.9) and 0.1 (−2.6, 2.8), respectively. ICC values of pooled LS and CS segments showed fair to good agreement and exhibited a similar trend (Table 4) with LS results showing higher ICC than CS.

**Table 4:** Intraclass correlation (ICC) of segmental longitudinal strain (LS) and circumferential strain (CS).

Parameter	Comparison	ICC (95% CI)	MAE	SDAE
LV LS	1.5T vs 0.55T	0.78 (0.69, 0.84)	2.4	1.9
LV CS		0.60 (0.49, 0.66)	3.1	2.7
RV LS		0.75 (0.61, 0.84)	1.7	1.3

LV LS	1.5T scan vs rescan	0.89 (0.85, 0.92)	1.8	1.4
LV CS		0.83 (0.77, 0.88)	2.4	2.1
RV LS		0.84 (0.74, 0.91)	1.5	1.1

LV LS	0.55T scan vs rescan	0.86 (0.81, 0.90)	1.6	1.3
LV CS		0.64 (0.52, 0.73)	2.6	2.4
RV LS		0.76 (0.59, 0.86)	1.3	1.3

*LV* left ventricle, *RV* right ventricle, *MAE* mean absolute error, *SDAE* standard deviation of the absolute error, *CI* confidence interval

**Table 5:** Longitudinal segmental strain Bland-Altman analysis.

	1.5T vs 0.55T (N =16)					1.5T repeatability (N = 10)					0.55T repeatability (N = 10)			
Segment	Bias^a^ (%)	STD	LOA (lower, upper)			Bias^b^ (%)	STD	LOA (lower, upper)			Bias^c^ (%)	STD	LOA (lower, upper)	
LV														
Pooled segments	0.8	2.9	−4.8	6.5		0.1	2.3	−4.4	4.6		−0.2	2.1	−4.3	3.9
Basal anterior	1.2	2.3	−3.3	5.8		0.3	2.0	−3.6	4.1		0.3	1.8	−3.2	3.8
Basal anteroseptal	1.1	3.4	−5.6	7.8		−0.6	1.5	−3.4	2.3		0.2	1.1	−1.9	2.4
Basal inferoseptal	0.1	2.1	−4.1	4.3		0.3	1.7	−3.0	3.5		−0.6	1.7	−4.0	2.9
Basal inferior	0.2	2.9	−5.5	6.0		−0.4	2.6	−5.5	4.7		−0.8	2.9	−6.4	4.8
Basal inferolateral	1.2	2.3	−3.3	5.6		0.2	2.0	−3.7	4.1		−0.8	2.2	−5.1	3.6
Basal anterolateral	1.0	2.6	−4.1	6.1		0.3	1.7	−2.9	3.6		0.7	2.3	−3.7	5.1
Mid anterior	1.2	2.9	−4.5	7.0		1.5	2.2	−2.8	5.8		0.3	2.3	−4.3	4.8
Mid anteroseptal	1.8	1.8	−1.7	5.4		0.6	1.3	−2.0	3.2		0.6	1.3	−2.0	3.3
Mid inferoseptal	1.7	2.4	−3.1	6.4		−0.4	1.5	−3.3	2.6		−0.4	1.2	−2.8	2.0
Mid inferior	1.0	3.6	−6.1	8.1		0.9	3.0	−5.0	6.8		−1.1	2.3	−5.5	3.4
Mid inferolateral	0.8	3.5	−6.1	7.6		2.0	2.4	−2.6	6.6		−0.6	1.7	−3.8	2.7
Mid anterolateral	0.1	3.4	−6.6	6.7		0.1	2.5	−4.8	5.0		0.4	1.9	−3.3	4.1
Apical anterior	1.0	3.0	−4.9	6.9		0.7	2.6	−4.5	5.8		0.6	1.8	−2.8	4.1
Apical septal	−0.8	3.6	−7.8	6.2		−1.3	1.6	−4.5	1.9		0.4	2.0	−3.5	4.3
Apical inferior	0.5	4.2	−7.7	8.7		−1.8	2.5	−6.7	3.1		−1.5	3.3	−8.0	4.9
Apical lateral	1.4	2.3	−3.2	5.9		0.1	2.8	−5.3	5.5		−0.7	2.1	−4.8	3.3
RV														
Pooled segments	0.2	2.1	−4.0	4.3		0.1	1.8	−3.4	3.6		0.4	1.8	−3.1	3.9
Basal anterior	0.2	2.7	−5.1	5.5		−0.1	1.4	−2.8	2.6		0.7	1.0	−1.3	2.7
Basal lateral	−1.0	1.4	−3.7	1.7		0.1	2.7	−5.2	5.4		1.1	2.6	−4.0	6.2
Basal inferior	0.1	1.7	−3.2	3.4		0.2	2.0	−3.7	4.1		0.3	2.3	−4.2	4.8
Mid anterior	1.5	1.9	−2.2	5.2		0.5	1.7	−2.8	3.8		−0.2	1.4	−2.9	2.5
Mid lateral	0.1	2.2	−4.2	4.4		0.2	1.2	−2.2	2.6		0.3	1.8	−3.2	3.8
Mid inferior	0.0	2.0	−3.9	3.9		−0.2	1.0	−2.2	1.8		0.1	1.4	−2.6	2.8

*STD* standard deviation, *LOA* limits of agreement, *LV* left ventricle, *RV* right ventricle

a Bias reported as mean difference of 1.5T-0.55T

b Bias reported as mean difference of 1.5T Scan 2 – 1.5T Scan 1

c Bias reported as mean difference of 0.55T Scan 2 – 0.55T Scan 1

Longitudinal and circumferential segment-by-segment reproducibility and repeatability are shown in Tables 5 and 6 . Overall, both measurements exhibited low mean bias; LS again tended to have narrower LoA than CS. For between scanner reproducibility, on a segment-by-segment level, the average strain bias and standard deviation were +0.83 (2.9) and +0.05 (3.9) for LV LS and CS, respectively. The LoA tended to be between −7 and +7 for lower and upper bounds; however, these limits were notably wider in some myocardial regions such as the apex and the inferior segments on the 2ch view (eg., −12, +10). 1.5T and 0.55T scan-rescan repeatability yielded similar findings with low biases at both field strengths, but narrower LoA than between scanner reproducibility. Levene’s test comparing repeatability at 1.5T vs 0.55T was non-significant for segmental LS (p = 0.36) and CS (p = 0.37), indicating comparable scan-scan repeatability of segmental strain measurements at 0.55T and 1.5T.

**Table 6:** Left ventricular circumferential segmental strain Bland-Altman analyses.

	1.5T vs 0.55T (N =16)					1.5T repeatability (N = 10)					0.55T repeatability (N = 10)			
Segment	Bias^a^ (%)	STD	LOA (lower, upper)			Bias^b^ (%)	STD	LOA (lower, upper)			Bias^c^ (%)	STD	LOA (lower, upper)	
Pooled segments	0.1	4.1	−8.0	8.1		0.2	2.3	−4.4	4.6		0.3	3.6	−6.7	7.2
3ch basal inferolateral	1.1	2.8	−4.5	6.6		−0.3	1.5	−3.3	2.7		−0.2	2.1	−4.4	4.0
3ch mid inferolateral	1.8	4.2	−6.3	10.0		1.0	1.5	−2.0	3.9		1.5	2.4	−3.3	6.3
3ch apical lateral	2.1	4.6	−6.8	11.0		0.9	4.2	−7.3	9.0		2.4	6.1	−9.7	14.4
3ch apical cap	1.2	3.2	−5.2	7.5		−0.1	2.5	−5.0	4.9		−0.6	2.9	−6.3	5.0
3ch apical anterior	−1.2	3.5	−8.0	5.6		0.7	2.4	−4.1	5.5		0.7	3.7	−6.5	7.9
3ch mid anteroseptum	0.5	4.1	−7.5	8.5		1.3	3.4	−5.3	7.9		1.1	2.3	−3.4	5.7
3ch basal anteroseptum	1.5	3.9	−6.1	9.2		3.7	4.9	−6.0	13.3		1.2	3.2	−5.0	7.5
4ch basal inferoseptum	−1.7	3.2	−8.0	4.6		1.3	2.7	−4.0	6.7		−0.5	3.4	−7.2	6.3
4ch mid inferoseptum	−0.8	3.3	−7.1	5.6		1.0	3.4	−5.6	7.6		0.4	3.9	−7.2	8.1
4ch apical septum	0.8	3.2	−5.6	7.1		0.4	2.8	−5.1	5.8		0.3	3.6	−6.7	7.3
4ch apical cap	1.1	4.1	−6.9	9.1		−0.3	2.1	−4.4	3.9		0.6	4.3	−7.8	8.9
4ch apical lateral	0.6	6.0	−11.2	12.3		−1.8	3.9	−9.4	5.8		0.2	5.4	−10.3	10.7
4ch mid anterolateral	−0.5	4.1	−8.4	7.5		−0.7	2.9	−6.3	4.9		−1.0	4.3	−9.5	7.4
4ch basal anterolateral	−0.9	3.2	−7.2	5.4		0.1	1.4	−2.7	2.9		0.5	4.1	−7.5	8.5
2ch basal inferior	−1.4	5.8	−12.8	10.0		0.0	3.4	−6.6	6.6		1.1	3.3	−5.3	7.5
2ch mid inferior	−0.7	6.5	−13.4	11.9		0.6	2.9	−5.1	6.2		−0.3	2.9	−6.0	5.5
2ch apical inferior	−0.7	5.1	−10.8	9.3		−0.3	3.2	−6.5	5.9		0.0	3.7	−7.2	7.3
2ch apical cap	0.6	2.8	−4.8	6.0		−2.5	4.2	−10.8	5.8		−1.5	3.1	−7.6	4.5
2ch apical anterior	0.5	3.6	−6.6	7.6		−2.6	4.2	−10.8	5.5		−0.5	3.6	−7.6	6.5
2ch mid anterior	−0.9	2.8	−6.3	4.5		−0.8	2.5	−5.7	4.2		0.1	1.9	−3.6	3.8
2ch basal anterior	−1.9	2.7	−7.1	3.3		0.4	2.8	−5.0	5.9		0.8	2.9	−4.9	6.5

*STD* standard deviation*, LOA l*imits of agreement*, 2ch* two chamber*, 3ch* three chamber, *4ch* four chamber

a BiasaFootnote aBias reported as mean difference of 1.5T-0.55T

b Bias reported as mean difference of 1.5T Scan 2 – 1.5T Scan 1

c Bias reported as mean difference of 0.55T Scan 2 – 0.55T Scan 1

**Fig. 3:**
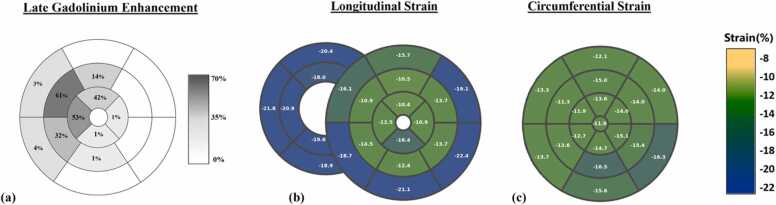
Polar plots of (a) averaged late gadolinium enhancement, (b) longitudinal, and (c) circumferential strain of six porcine hearts 8 weeks after myocardial infarction. For each segment shown in (a), the percentage area within each segment with LGE (0–100%) was measured and averaged over six pigs

**Fig. 4:**
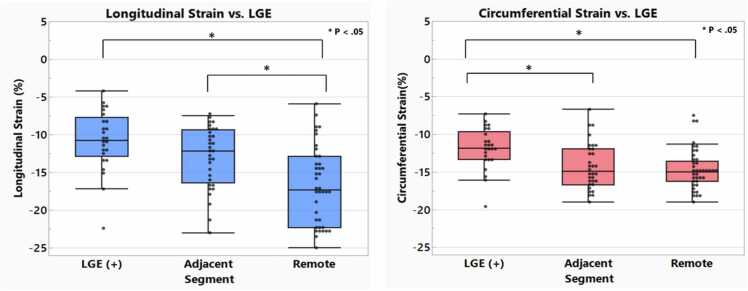
Segmental strain findings based on late gadolinium enhancement (LGE) status in six porcine hearts 8 weeks post myocardial infarction. Segments defined as having any amount of LGE (LGE+); adjacent to an LGE+ segment, and remote. Error bars denote standard deviation

LGE imaging at 8 weeks post-MI in six pigs showed infarct scarring primarily in the anterior and septal segments of the mid and apical short-axis slices (Fig. 3). Segmental LS in LGE+ vs remote segments was −10.8% ± 4.0 vs −16.8% ± 5.1, p < 0.001. Segmental LV CS in LGE+ vs remote segments was −11.9% ± 2.7 vs −14.6% ± 2.7, p = 0.001. These findings are displayed in Fig. 4.

## 4 Discussion

We evaluated the feasibility of SENC at 0.55T and compared results to 1.5T utilizing a segmented gradient echo sequence with comparable implementations on both systems. The primary concern for SENC at lower field strengths is the significant reduction in SNR. The Rician noise distribution in magnitude-reconstructed magnetic resonance imaging (MRI) introduces a bias that could potentially impact SENC-based strain measurements. Additionally, myocardial T1 is shorter at lower field (701 ms at 0.55T vs 955 ms at 1.5T) [10], reducing the persistence of the tagging signal compared to 1.5T. However, the shorter T1 may have minimal impact on peak systolic strain measurement, the parameter of primary interest. Peak strain occurs early in the cardiac cycle within about 300 ms of the tagging pulse, which is applied at the R-wave.

Despite these technical challenges, low-field SENC on a commercially available 80 cm wide-bore 0.55T system yielded global strain measurements in healthy measurement that were congruent with those acquired at 1.5T. Globally, LV GLS, LV GCS, and RV GLS were consistent with normative values [11] and did not demonstrate a significant difference between field strengths. While reproducibility between 1.5T and 0.55T showed little mean bias, LoA were at times broad, larger than those observed for the scan-rescan repeatability, which was generally similar at both field strengths. Further, when adjunctive SENC imaging was added to a porcine investigation of MI, we observed the expected impairment in segmental LS and CS in scar segments compared to remote segments.

Our findings align with previous studies that have demonstrated the feasibility of low-field MRI for various applications. For instance, Varghese et al. showed that comprehensive CMR exams could be effectively conducted on a 0.55T system [4]. Similarly, Campbell-Washburn et al. highlighted the potential of low-field MRI for cardiac imaging, emphasizing the need for optimized sequences to mitigate the reduced SNR [10]. These studies support our observation that low-field systems can provide diagnostic-quality images, albeit with some limitations in SNR and spatial resolution.

Across our study’s schema, LS proved more reproducible than CS, in agreement with echocardiography literature [12]. Global strain also demonstrated better reproducibility than segmental findings, similarly observed in a meta-analysis of cardiac strain derived from feature-tracking techniques [13]. LV LS and CS demonstrated minimal systematic bias for both scan-rescan repeatability and reproducibility between 1.5T and 0.55T, with the latter generating wider LoA. This held true for both global and segmental findings. This suggests that while the mean difference in strain between 1.5T and 0.55T for a group of participants may be near zero, variation in findings between 1.5T and 0.55T can exist for individual subjects, and that it may not be possible to use strain measurements interchangeably across field strengths. Overall, LV GLS and GCS reproducibility between field strengths and scan-rescan repeatability for both 1.5T and 0.55T were similar to the previously reported interstudy reproducibility of GLS and GCS conducted at 1.5T, small systematic biases and LoA of ±3–4% [5], [7]. Erley et al. compared intervendor reproducibility at three separate 3T centers, which may better serve as proxy to our 1.5T vs 0.55T comparison, and reported GLS and GCS reproducibility wider than what we observed, with biases ranging from 0.6–1.88% with LoA of ±3–7% [14].

On the segmental level, pooled segmental LS and CS ICC values generally showed moderate to good reliability [15]; notably, LV GCS showed poorer agreement between field strengths (ICC = 0.6) and for 0.55T scan-rescan (0.64) repeatability. With these exceptions, our findings are similar to the reported pooled ICC segmental LS (0.84) and CS (0.85) findings by Bucius et al. [7]. To our knowledge, ours is the first study to evaluate reproducibility and repeatability of SENC on a segment-by-segment basis. Bland-Altman analysis demonstrated larger but still reasonable biases (0.1% to 2.1%) between field strengths for both LS and CS, indicating minimal systematic differences at the segmental level between 1.5T and 0.55T. However, the LoA were at times substantial, suggesting wide variability in segmental strain for the same patient on different systems. This variability was especially potent for CS and the at apical and inferior regions of the heart. Scan-rescan segmental agreement at 1.5T and 0.55T also demonstrated low bias, with smaller standard deviation and tighter LoA when compared to reproducibility between systems, as expected. The size of this difference, however, was not profound, with notably broad LoA still present especially for CS at 0.55T. This suggests that the strain differences we observed between field strengths may have at least in part been due to significant scan-scan variability at both field strengths. Based on these results, LV CS may lack sufficient reliability in its current implementation for use at 0.55T. We are continuing to explore other pulse sequence strategies, including spiral readouts, that may improve this performance in the future.

In a porcine model of MI, imaging at 8 weeks post-MI showed fibrosis, evidenced by LGE concentrated in the mid to apical, anterior, and septal segments, consistent with the ligated LAD territory. Impaired longitudinal and circumferential segmental strain correlated with this scar pattern. Our findings are similar to previous studies at higher field evaluating induced MI of the porcine heart using CMR strain [16], suggesting that SENC at 0.55T is also capable of detecting segmental strain differences. Comparing infarcted vs remote American Heart Association segments, LS exhibited a larger difference in strain between segments than CS. Remote segments showed mostly preserved longitudinal function, while CS was more globally reduced across the porcine heart. While this finding is not surprising, it does demonstrate the capability for SENC at 0.55T to detect regional pathological differences in myocardial strain.

## 5 Limitations

The human subjects included only a small cohort of relatively young, healthy subjects, but showed no preliminary evidence of significant differences in strain results compared to 1.5T. SENC was not acquired in the porcine models before infarction, and therefore the regional differences observed after infarction could be confounded by baseline differences.

## 6 Conclusion

In summary, our study demonstrates the feasibility of using SENC at 0.55T, showing comparable global strain measurements to those obtained at 1.5T, laying a foundation for the application of strain imaging in low-field CMR. The repeatability of global and segmental LS at 0.55T was similar to established 1.5T performance; however, CS reproducibility was notably poorer. Segmental strain reproducibility showed little systematic bias but rather large LoA. However, the ability to detect segmental strain differences in an animal model of MI further supports the potential clinical utility of low-field SENC. Further work is needed to establish normative values of SENC on the low-field scanner and to improve CS reliability.

## Funding

Research reported in this publication was supported by the 10.13039/100000050National Heart, Lung, and Blood Institute of the 10.13039/100000002National Institutes of Health under award numbers R01 HL161618 and R01 HL167912. Additionally, this work was supported in part by The Ohio State University Keenan Center for Entrepreneurship Accelerator Award. O.P.S. is supported by the Robert F. Wolfe and Edgar T. Wolfe Foundation, Columbus, Ohio.

## Author contributions

**Juliet Varghese:** Writing – review & editing, Visualization, Validation, Supervision, Software, Resources, Project administration, Methodology, Investigation, Data curation, Conceptualization. **Yuchi Han:** Writing – review & editing, Visualization, Supervision, Software, Resources, Project administration, Methodology, Investigation, Formal analysis, Data curation, Conceptualization. **John L. Heyniger:** Writing – review & editing, Writing – original draft, Formal analysis. **Vedat O. Yildiz:** Methodology, Formal analysis. **Preethi Chandrasekaran:** Formal analysis. **Katherine Binzel:** Writing – review & editing, Supervision, Methodology, Data curation, Conceptualization. **Orlando P. Simonetti:** Writing – review & editing, Visualization, Validation, Supervision, Software, Resources, Project administration, Methodology, Investigation, Funding acquisition, Formal analysis, Data curation, Conceptualization. **Yingmin Liu:** Writing – review & editing, Formal analysis, Data curation. **Nikita Nair:** Formal analysis. **Donel Tani:** Formal analysis. **Farouk Osman:** Formal analysis. **Vinay Kumar:** Data curation, Conceptualization. **Shyam S. Bansal:** Data curation, Conceptualization.

## Declaration of competing interests

The authors declare the following financial interests/personal relationships which may be considered as potential competing interests. Orlando Simonetti receives institutional research support from Siemens and Myocardial Solutions. Donel Tani and Farouk Osman are employees of Myocardial Solutions.
